# Computer Vision Syndrome Among Health Sciences Students in Saudi Arabia: Prevalence and Risk Factors

**DOI:** 10.7759/cureus.7060

**Published:** 2020-02-20

**Authors:** Abdullah Altalhi, Waleed Khayyat, Osama Khojah, Mohammed Alsalmi, Hashem Almarzouki

**Affiliations:** 1 Medicine, King Saud Bin Abdulaziz University for Health Sciences, Jeddah, SAU; 2 Ophthalmology, King Abdulaziz Medical City, Ministry of National Guard, Jeddah, SAU

**Keywords:** computer vision syndrome, college students, medical students, dry eyes

## Abstract

Introduction

Computer vision syndrome (CVS) is defined as a group of vision-related symptoms that result from the continuous use of devices with digital displays, such as computers, tablets, and smartphones. Students nowadays can find resources and books online on their smartphones easily, hence, reducing the use of paper-based reading materials. This might lead to a number of ocular symptoms. In this study, we aim to assess the prevalence and determine the risk factors of CVS among students at King Saud Bin Abdulaziz University for Health Sciences (KSAUHS) in Jeddah.

Materials and methods

This is an observational descriptive cross-sectional study design. Students of Colleges of Medicine, Applied Medical Sciences, and Science and Health Professions at KSAUHS were asked to fill an electronic self-administered survey. The survey instrument included questions on demographic information, digital devices using habits, frequency of eye symptoms, and ergonomic practices.

Results

The sample size was 334 students, 55% of whom were males. The most used device was the mobile phone (78%), and the most common reason for using an electronic device was for entertainment (80%). The frequency of reported eye symptoms was as follows: headache (68%), feeling of an affected eyesight (short- or long-sightedness (65%)), eye itchiness (63%), burning sensation (62%), excessive tearing (58%), unclear vision (52%), redness (51%), dryness (48.3%), photophobia (47%), painful eye (44%), foreign body sensation (40%), excessive blinking (40%), difficulty in focusing on near objects (31%), halos around objects (28%), double vision (21%), and difficulty moving eyelids (9%). The most commonly applied ergonomic practice was adjusting display brightness based on the surrounding light brightness (82%). The rest of the ergonomic practices were less applied as follows: taking breaks while using the device (66%), sitting with the screen on face level (59%), sitting while the top of the screen on eye level (43%), sitting with the screen more than 50 cm away (32%), using antiglare filter (16%). The number of eye symptoms reported was significantly greater in female students (using Mann-Whitney U test) (U= 11056.500, p= 0.002), students who wear glasses (U= 11026, 0.002), and students who observe glare on their screens (U= 8363, p= 0.043).

Conclusion

CVS symptoms are commonly reported among health sciences students who use different electronic devices. The occurrence of CVS symptoms was significantly higher among female students, those who observe glare on screens, and those who wear eyeglasses. However, long duration of device use was not significantly associated with increased CVS symptoms. Ergonomic practices are not usually applied by most of the students, which necessitates more efforts to increase their awareness of the correct way of using devices.

## Introduction

Computer vision syndrome (CVS), also referred to as digital eye strain, is defined as a group of vision-related and muscular symptoms that result from continuous use of devices with digital displays, such as computers, televisions, tablets, and smartphones. This syndrome started to arise after the usage of computers in the mid-twentieth century [1]. With the help of technological progress, devices nowadays are essential in every house, office, and even pockets in the form of smartphones, as most of the things done by computers can be currently done by smartphones, explaining the excessive use of mobiles. The general population, specifically students, can find resources and books online on their smartphones easily, making them reduce the use of paper-based reading materials. In addition, some jobs require continuous staring at the computer screen for hours on a daily basis. This will mostly lead to a number of symptoms related to the vision and probably musculoskeletal issues. CVS symptoms include eye strain, headache, blurry vision, dry eyes, and pain in the shoulders or neck. CVS is a common health problem and the majority of computer users are affected by it. However, it does not appear to be too strenuous that it drives them to the doctor’s office [2]. Many studies showed a high prevalence of CVS among office workers and college students. In 2016, one study found a prevalence of 67.4% among computer office workers with the disease being more common in females [3]. Another study, in 2014, was done on 416 engineering and medical students in India with a prevalence of 81.9% and 78.6%, respectively [4]. Moreover, bankers had a study done on them in 2016, showing a prevalence of 73% and the presence of some risk factors such as poor room lighting and improper distance between the eye and the screen [5]. Therefore, in this study, we aim to assess the prevalence and determine the risk factors of CVS among students at King Saud Bin Abdulaziz University for Health Sciences (KSAUHS) in Jeddah.

## Materials and methods

Study population and sampling

The study was conducted at KSAUHS, Saudi Arabia. 334 students from the College of Medicine, Applied Medical Sciences, and Science and Health Professions at KSAUHS were asked to fill electronic self-administered questionnaires. The sample size was calculated using the prevalence of previous studies (70%). The study was conducted from March 2018 to July 2019 with the data collection conducted during April 2018. Verbal consent was acquired from all of the participants after explaining the confidentiality measures and the objectives of the research. Convenient sampling technique was used to collect the data, and all students who were willing to participate were included. Ethical approval was obtained from the ethics review committee of King Abdullah International Medical Research Center (KAIMRC), Saudi Arabia. Confidentiality of data was maintained thoroughly as no personal information was obtained and all questionnaires were kept anonymous. 

Study instruments

An electronic self-administered questionnaire was used to collect demographic data, symptoms of CVS, and data about computer/electronic device usage. Demographic data collected include age, gender, the college that the participant attends, the electronic device used the most, and the main purpose of using the said device. Symptoms of CVS included the presence of pain in and around the eyes, burning sensation in the eye, sore/irritated eyes, sensation of foreign body, excessive tearing, twitching of eyelids, difficulty moving the eyelids, eye dryness, headaches, blurred near vision, blurred distant vision, red eyes, double vision, and changes in visualizing colors. The participants were also asked to report the frequency by marking one of the following - never, rarely, often, or most of the time. Moreover, the data collected on computer/electronic device usage were daily computer usage in hours, whether or not rests in between were taken and the length of that rest, the position of the device, usage of glasses or contact lenses, usage of anti-glare filters, and any chronic condition which affects them. The aforementioned questions were used to assess the risk factors based on the literature review done on CVS. The data collection sheet was provided to participants in Arabic but then was translated into English for data entry and analysis.

Statistical methods and variables 

Data were entered using Microsoft Excel 2017 and analyzed by Statistical Package for the Social Sciences (SPSS) version 22. For the analysis, descriptive statistics and Mann-Whitney U test were performed with a confidence level of 95%. The main outcome was CVS which is defined as a combination of eye and vision problems associated with the continuous use of computers, tablets, e-readers and cell phones [6]. A p-value of <0.05 was taken as significant.

## Results

A total of 334 students were included in the study. The median age of the participants is 20 years of age (interquartile range= 2). 55.4% (185) of the sample were males. The most commonly used device was the mobile phone (78%) with the main goal of using that device was entertainment (80%). 97.3% (325) of the study participants had at least one symptom of CVS. The symptoms most experienced were headache (68%), feeling of affected eyesight (i.e. temporary long or short-sightedness) (65%), and itchy eyes (63%); whereas, the symptom least experienced was difficulty moving the eyelids (9%) (Table 1). The number of eye symptoms was significantly greater in female students (184 VS 149, p=0.002) and students who wear glasses (p=0.002). The ergonomic practices most applied were adjusting the brightness based on the surrounding lighting (82%), taking breaks while using the device (66%), and having the screen on face level (59%); whereas, the least applied practice was using anti-glare filters (16%) (Table 2). The number of CVS symptoms was significantly greater in students observing glare on the screen (p=0.004). Ergonomic practices were not associated with a decreased number of symptoms (p= 0.947). Moreover, longer duration of usage (>6 hours) is not significantly associated with an increase in CVS symptoms (p= 0.689).

**Table 1:** Frequency of symptoms among participants

Symptom	Frequency (%)	Symptom	Frequency (%)
Headache	68%	Photophobia	47%
Feeling of an affected eyesight (temporary long or short-sightedness)	65%	Pain around the eye	44%
Itchy eye	63%	Foreign body sensation	40%
Burning sensation	62%	Excessive eye blinking	40%
Tearing	58%	Difficulty focusing on near objects	31%
Unclear vision (blurry)	52%	Halos around objects	28%
Red eyes	51%	Double vision	21%
Dryness	48%	Difficulty moving eyelids	9%

**Table 2:** Frequency of ergonomic practices

Ergonomic practice	Frequency (%)
Adjusting brightness based on the surrounding lighting	82
Taking breaks while using the device	66
Having the screen on the level of the face	59
Sitting while the top of the screen is on the eye level	43
Having the screen more than 50cm away	32
Using anti-glare filters	16

## Discussion

This study aimed to assess the prevalence of CVS and determine the risk factors among students of different colleges at KSAUHS in Jeddah, Saudi Arabia. Also, to determine the prevalence of the application of ergonomic practices during the use of the devices. Previous studies of CVS have shown heterogeneity in their results which can be attributed to the varying research methodologies. In our research, out of a total of 334 participants, 97.3% (325) reported at least one symptom. Compared to previous studies that found a prevalence of 67.4% among computer office workers in Sri-Lanka, 72% in the United Arab Emirates (UAE), among computer using university students, 80.3% in the Indian city of Chennai among medical and engineering university students, and 89.9% of five Malaysian universities students experience at least one of the CVS symptoms [3,4,7,8]. Our study showed a higher prevalence of CVS that may be attributed to the relatively small sample size. In our study, the most commonly reported symptom was headache (68%), followed by a feeling of affected eyesight (i.e. temporary long or short-sightedness) (65%). Headache was the most common reported CVS symptom across multiple studies [3,8,9]. Multiple factors come into play when it comes to headache; some studies pointed out that the visual fatigue increases as the distance shortens between the individual and the device, also some have mentioned that the constant shifting and accommodating that the eye and the extraocular muscles endure for a very long period of time causes a stress on the muscles and fatigues the eye which eventually leads to the headache [3,7]. The prevalence of symptoms was higher in our study when compared to other studies. For example, while comparing university students in the UAE and medical and engineering university students in Chennai, the reported prevalence of headache was 53.3% and 43.2%, burning sensation 54.8% and 32.3%, and blurry vision 24.8% and 16.4%, respectively [4,7]. The differences between the two genders remain constant across multiple studies, as females display a significantly greater number of CVS symptoms [7,10,11]. Nevertheless, males were reported to have a higher prevalence in some individual symptoms like burning sensation, dry eyes, red eyes, and blurred vision [4].

Multiple studies have reported that participants experienced more pronounced symptoms after using the device for more than six hours or spending more than seven hours a day on the computer which was a significant predictor of CVS [2,12,13]. However, we found that there is no association between the usage of the device for more than six hours and an increase in the number of symptoms. In accordance with previously conducted studies, we found that the number of symptoms significantly increases in those wearing eyeglasses [2,4]. A possible explanation might be that the computer screens are formed by pixels instead of solid images and that computer work requires close proximity to the device means that the eyes must work harder in those with corrective issues just to keep the images in focus [4]. The main limitations of our study were that it is a cross-sectional study conducted using convenient sampling involving students of a single university and that our study did not include an ophthalmic examination; the symptoms reported were self-reported.

## Conclusions

CVS symptoms are commonly reported among health sciences students who use electronic devices. The frequency of CVS symptoms was significantly higher among female students, those who observe glare on the screens, and those who wear eyeglasses. On the other hand, increased duration of device use was not significantly associated with a higher frequency of CVS symptoms. It was also found that ergonomic practices are not usually applied by most of the students. Therefore, more efforts should be directed toward educating the students on the correct way of using electronic devices.
